# Memantine Derivatives as Multitarget Agents in Alzheimer’s Disease

**DOI:** 10.3390/molecules25174005

**Published:** 2020-09-02

**Authors:** Giambattista Marotta, Filippo Basagni, Michela Rosini, Anna Minarini

**Affiliations:** Department of Pharmacy and Biotechnology, Alma Mater Studiorum-University of Bologna, Via Belmeloro 6, 40126 Bologna, Italy; giambattista.marott2@unibo.it (G.M.); filippo.basagni2@unibo.it (F.B.); michela.rosini@unibo.it (M.R.)

**Keywords:** memantine, Alzheimer’s disease, multi target directed ligand, hybrid structures

## Abstract

Memantine (3,5-dimethyladamantan-1-amine) is an orally active, noncompetitive N-methyl-D-aspartate receptor (NMDAR) antagonist approved for treatment of moderate-to-severe Alzheimer’s disease (AD), a neurodegenerative condition characterized by a progressive cognitive decline. Unfortunately, memantine as well as the other class of drugs licensed for AD treatment acting as acetylcholinesterase inhibitors (AChEIs), provide only symptomatic relief. Thus, the urgent need in AD drug development is for disease-modifying therapies that may require approaching targets from more than one path at once or multiple targets simultaneously. Indeed, increasing evidence suggests that the modulation of a single neurotransmitter system represents a reductive approach to face the complexity of AD. Memantine is viewed as a privileged NMDAR-directed structure, and therefore, represents the driving motif in the design of a variety of multi-target directed ligands (MTDLs). In this review, we present selected examples of small molecules recently designed as MTDLs to contrast AD, by combining in a single entity the amantadine core of memantine with the pharmacophoric features of known neuroprotectants, such as antioxidant agents, AChEIs and Aβ-aggregation inhibitors.

## 1. Introduction

Alzheimer’s disease (AD) is a chronic neurodegenerative condition that slowly destroys nerve cells, leading to a progressive cognitive decline. As for major chronic diseases, a pluralism of causative features is thought to be implicated, resulting in complex networks of perturbation. The mechanistic understanding of synaptic damage might offer a valuable key for developing future therapeutic strategies to tackle AD progression. In this context, excitotoxicity is emerging as a pivotal event. Caused by excessive or prolonged glutamate exposure, this pathological condition alters neurons to death.

N-methyl-D-aspartate (NMDA) receptors (NMDAR) are ionotropic glutamate receptors primarily involved in synaptic plasticity underlying learning and memory. However, they are also prime actors of excitotoxic damage occurring during chronic neurodegenerative injuries [1]. Increasing consensus indicates that functional properties of NMDAR are governed by their localization, with synaptic NMDAR (sNMDAR) contributing to cell plasticity and neurotrophic processes, while extrasynaptic NMDAR (eNMDAR) triggering apoptotic signalling pathways [2]. This effect accounts for the difficulties encountered in the design of safe and effective cures acting on NMDARs, and contemporary suggests that a selective targeting of the eNMDARs represents a promising approach to treat neurodegenerative conditions [3].

Memantine is an uncompetitive NMDAR antagonist approved to treat moderate-severe AD patients. It acts as an open-channel blocker with a relatively rapid off-rate from the channel [4]. Due to these peculiar kinetics, memantine mainly enters the channel in conditions of excessive and prolonged glutamate exposure, preferentially acting on extrasynaptic/tonically-activated NMDAR over synaptic/phasically-activated NMDAR [5,6]. This peculiar profile, which allows memantine to contrast excitotoxicity while preserving glutamatergic synaptic functioning, possibly accounts for the clinical tolerability of the drug [7]. Unfortunately, however, memantine offers only palliative benefits to patients. Possibly, the explanation for this issue might be the complex neurotransmitters dysfunction characterizing AD, which suggests the modulation of a single neurotransmitter system to be inadequate to face the disease. Increasing evidence indicates a mutual feedback connection between the glutamatergic and the cholinergic systems, which both possess strong implications in cognitive functions [8]. Furthermore, the failure of calcium homeostatic balance deriving from NMDAR overactivation as well as metal dyshomeostasis (Fe, Zn, Cu) [9,10] orchestrates several pathologic features, including oxidative damage [11,12], neuroinflammation and protein misfolding and aggregation [13,14], all contributing to the neurotoxicity mediated by excitotoxic damage. Indeed, the presence of neuritic plaques constituted mainly by insoluble Aβ-peptide (Aβ) in the brain parenchyma, is one of the hallmarks required for AD diagnosis.

**Figure 1:**
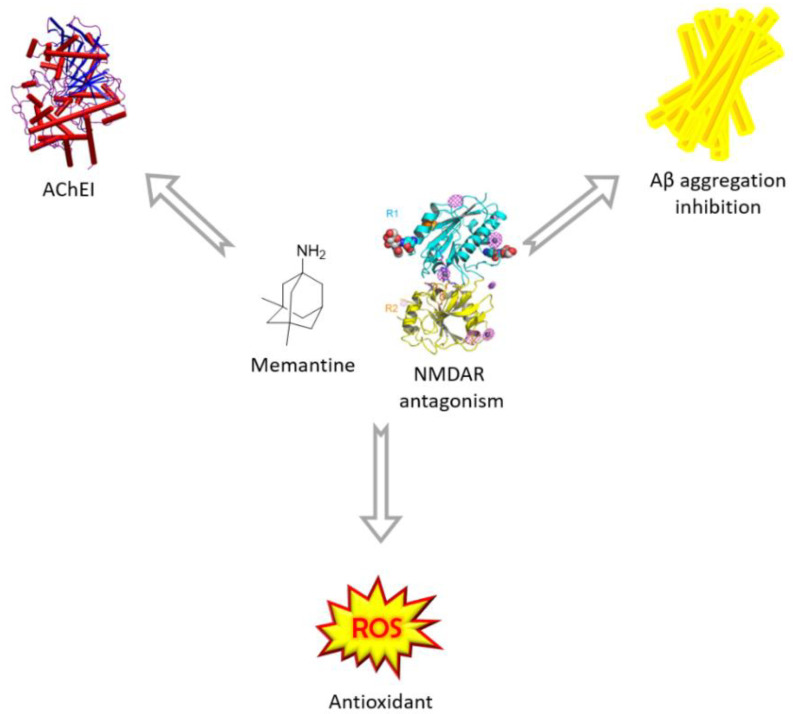
Conjugation strategies exploiting memantine’s NMDAR antagonism to tackle AD.

On this basis, research efforts have recently focused on the identification of therapeutic tools that could target glutamatergic hyperactivation and excitotoxicity-related mechanisms, triggering a synergistic response [15]. Thus, Multi Target Directed Ligands (MTDLs), i.e., single molecules acting on different targets simultaneously, are emerging at a rapid pace as a valuable opportunity to restore the complex interplay among multiple NMDAR-mediated alterations [16,17]. In this context, memantine is viewed as a privileged NMDAR-directed structure, and therefore, represents the driving motif in the design of a variety of multifunctional compounds. In the search for a disease-modifying drug for AD, the MTDL approach has been applied by different research groups to obtain hybrid compounds able to hit simultaneously different pathways implicated in AD [18]. In this review, we present selected examples of small molecules designed by merging in a single entity the amantadine core of memantine with the chemical features of known neuroprotectants performing through different mechanism of action, such as antioxidant and anti-aggregating activity or AChE and monoaminoxidase inhibition (Figure 1).

## 2. Memantine and Cholinesterase Inhibitor Hybrids

To date, only two classes of drugs are licensed for the treatment of AD: AChEIs for mild to-moderate disease and memantine for moderate and severe disease. AChEIs prevent cholinesterase enzymes (ChEs) from degrading acetylcholine (ACh), in order to increase the synaptic concentration and the duration of action of the neurotransmitter [19].

Among the various theories that try to explain the characteristic etiopathological cascade of AD, one of the most investigated is the cholinergic hypothesis. Indeed, cholinergic loss is one of the driving impairments since the early symptoms of the disease, unavoidably leading to the characteristic cognitive decline [20]. Actually, the standard-of-care treatment for AD is the combination of cholinesterase’s inhibitors, with the aim to potentiate the cholinergic transmission, and memantine [16]. AChEIs currently used in AD therapy, such as Rivastigmine, Donepezil and Galantamine, are able to stabilize cognitive decline for 3 to 6 months, but do not halt or slow down the progression of the disease [19,21]. Also, some Donepezil-based derivatives prevent the formation of amyloid plaques, since a connection has been demonstrated between AChE activity and plaque formation [22]. Indeed, Aβ peptide interacts with the peripheric anionic site (PAS) of human AChE (*h*AChE), that operates as a nucleation site enhancing Aβ aggregation [23].

Moreover, throughout AD progression there is a reduction of AChE levels and an increase of those of a nonspecific cholinesterase, butyrylcholinesterase (BChE); thus, targeting the latter could result in higher therapeutic efficacy [20]. Recently, some novel compounds bearing a carbamate moiety have proved to selectively bind to BChE and to act as neuroprotectors in vivo, pointing out a potential advantage for a disease-modifying therapy [24].

Following the polypharmacological approach and the clinical relevance of both memantine and AChEIs, several MTDLs have been developed by joining memantine to pharmacophores, inhibiting ChE with the aim to synergistically and more effectively tackle the AD pathological cascade. Herein there are reported a series of these hybrids divided by their ChEI core.

### 2.1. Tacrine-Adamantanes Hybrids

**Figure 2:**
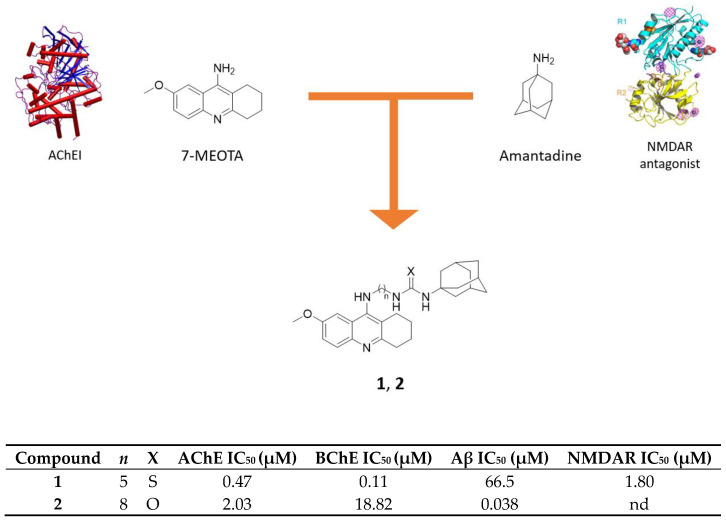
Drug design and biological activities of 7-MEOTA-amantadine hybrids **1** and **2**.

Tacrine (THA) is a non-competitive reversible AChEI active in the central nervous system (CNS) and slightly selective for BChE [25]. It was the first AChEI drug approved by FDA to combat AD, but it was withdrawn from the market due to its hepatoxicity [26]. Its derivatives 7-methoxytacrine (7-MEOTA) and 6-chlorotacrine (6-Cl-THA) are potent anti-ChE compounds with lower side effects [27,28]. An interesting strategy to obtain a multimodal profile towards ChE and NMDAR is to combine tacrine scaffold with the adamantane core through tethers of different length. In this view, hybrids of 7-MEOTA and amantadine, linked by methylene-thiourea or methylene-urea tethers, were designed and synthesized. The new compounds showed micromolar to submicromolar activities towards *h*AChE and human BChE (*h*BChE), NMDA receptor and Aβ_1-40_ aggregation (Figure 2) [29,30].

These hybrids act as competitive inhibitors of *h*AChE and *h*BChE with thioureas displaying higher selectivity for the latter; this may be beneficial, since it has been demonstrated that the levels of this enzyme increase in the course of AD (Figure 2) [20]. They have lower IC_50_ values for both enzymes than reference 7-MEOTA (10.50 μM for *h*AChE and 21 μM for *h*BChE), with the hybrids bearing a five-carbon linker that are the most potent in both series, suggesting that this is the optimal distance for target interaction [29,30]. Furthermore, the urea derivative **2** showed the highest inhibition of Aβ_1-40_ aggregation, while the thiourea **1** inhibits NMDAR with a similar IC_50_ value as memantine (1.80 μM vs. 1 μM) and the same efficacy as open channel blocker, but with slower kinetics, which may suggest why it preferentially binds to eNMDAR [31]. In fact, selectivity for eNMDARs is highly desirable due to their involvement in glutamate-mediated excitotoxicity and it is achieved through use-dependent inhibitors with a slow onset of action [32]. In addition to these activities, most of the compounds tested displayed weak-to-moderate activity towards BACE-1 enzyme, which, alongside inhibitory activity on Aβ_1-40_ aggregation previously showed, proves their potential antiamyloid properties.

Molecular modelling studies performed on compounds **1** and **2** showed how the 7-MEOTA moiety binds to the catalytic anionic site (CAS) while the amantadine core binds to the PAS of both cholinesterases. In *h*AChE, the 7-MEOTA scaffold creates π-π interactions within the cation-π site, while the methoxy group stabilizes the complex through hydrogen bonding. The alkyl chain contributes to stabilize the molecule-enzyme complex through the interaction of phenyl groups of amino acid residues located in the middle of the gorge, whereas the amantadine moiety establishes π-aliphatic interactions and several Van der Waals interactions at the edge of the gorge in the PAS. In the *h*BChE, the interactions are similar, with the nitrogen atom of the three-fused ring structure and the secondary amino group that create further hydrogen bonding. Based on the results obtained from docking studies, assumptions were made also to understand the ability of these hybrids to counteract Aβ_1-40_ aggregation; it was supposed that the three-ring structure of 7-MEOTA establishes π-π interactions with the hydrophobic regions of Aβ, while the adamantane cage is involved in non-aromatic interactions. Most urea derivatives studied here are superior than their thiourea counterparts in inhibiting amyloid fibrillization, probably due to the urea moiety that creates stronger hydrogen bonds.

**Figure 3:**
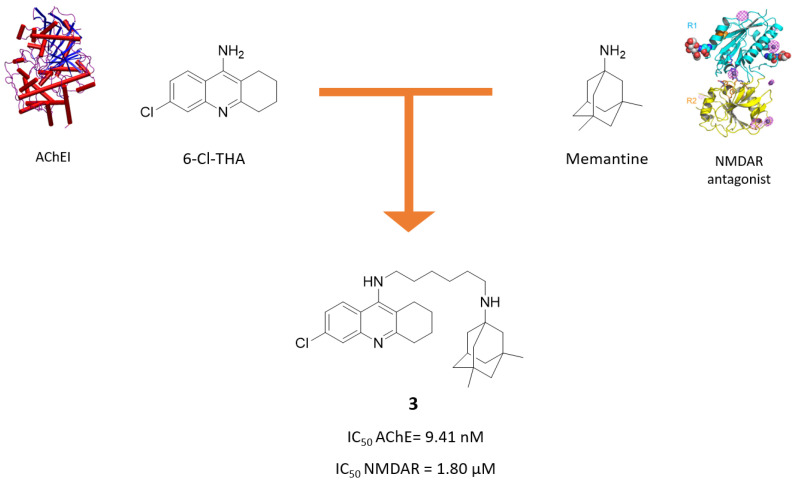
Drug design and biological activities of 6-chlorotacrine-memantine hybrid **3**.

Following a similar strategy, Kaniakova, M. and coworkers designed and synthesized the 6-Cl-THA-memantine hybrid **3** (Figure 3) [33]. The esamethylene linker was chosen based on the optimal spacer previously found in a series of galantamine-memantine hybrids, which will be discussed later.

Compound **3** demonstrated to act in a multimodal fashion, with a good activity towards AChE and NMDAR, as well as a higher neuroprotection ability than memantine after microinjection of NMDA into the dorsal rat hippocampus. In particular, **3** turned out to be more potent than parent compound 6-Cl-THA in inhibiting AChE (IC_50_ = 9.41 nM vs. 20 nM), but less potent than memantine in blocking NMDAR (IC_50_ = 1.80 μM vs. 0.79 μM). Like memantine, it targets the GluN1/N2B subunit, acting as an open channel blocker. Due to its promising pharmacological properties, its ability to cross the blood-brain barrier (BBB) was predicted using the PAMPA assay and its cytotoxic effect in CHO-K1 mammalian cells was assessed. Results suggest that it has a high probability of passing the BBB (Pe = 9.20 × 10^−6^ cm s^−1^), but is more cytotoxic than 6-Cl-THA (IC_50_ = 5.93 μM vs. 70.7 μM).

**Figure 4:**
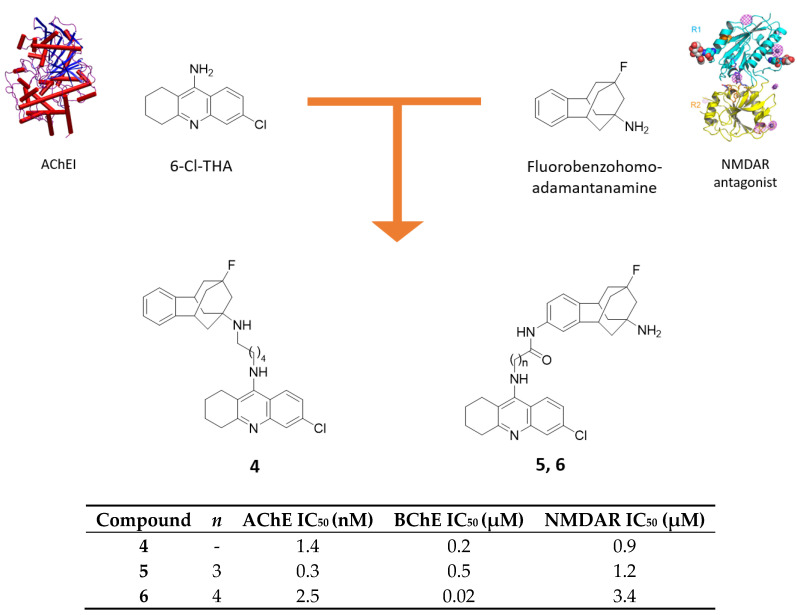
Drug design and biological activities of 6-chlorotacrine-benzohomoadamantane hybrids **4**–**6**.

Beside adamantylamine scaffold, its benzofused analogs have been extensively studied as NMDAR antagonists, with fluorobenzohomoadamantanamine being more potent than memantine and amantadine [34]. Due to its promising activity, Perez-Areales and coworkers combined this scaffold with 6-Cl-THA moiety through different linker lengths and in different linkage position to evaluate the potential pharmacological value of hybrids formed in this way (Figure 4) [35].

The resulting hybrids are better inhibitors of *h*AChE than 6-Cl-THA (IC_50_ = 0.3–2.5 nM vs. 14.5 nM). In particular, with a primary amino group attached to the adamantane moiety, the activity is augmented, with compound **5** being the most potent; the importance of the unsubstituted amino group is prominent in the inhibition of *h*BChE, for which hybrid **6** has the lowest IC_50_ value. However, only few of the tested hybrids turned out to be more potent than 6-Cl-THA (0.5 μM) in inhibiting *h*BChE.

Compounds **4** and **5** resulted as the most potent against NMDARs. Unlike in memantine, where the methylation of the amino group leads to a reduced activity, here the monomethylation of the bridgehead amino group in the polycyclic scaffold is tolerated and both compounds are about 2-fold more potent than benzohomoadamantanamine (IC_50_ = 0.9 and 1.2 μM vs. 1.9 μM). Particularly, in nitrogen-connected series, the hybrid with the longer linker has the best activity, while in the benzo-attached series, the opposite pattern is observed. Apart from the discussed targets, the compounds showed negligible inhibitory efficacy on BACE-1 enzyme and on Aβ_42_ and tau aggregation. Finally, PAMPA assay for brain permeation predictions was carried out with the results showing a more efficient BBB permeation for derivatives bearing a substituted amino group in the benzofused core (**4**), probably due to their higher lipophilicity. These studies show that hybrid molecules bearing an aminoadamantane moiety bound to THA or one of its derivatives exert their action in various pathways involved in AD in low-micromolar or nanomolar ranges [35]. In many cases, the spacer plays an important role in modulating the activity: the proper spacers’ length and nature is fundamental to the optimal interaction of pharmacophores to their respective binding sites.

### 2.2. Galantamine-Memantine Hybrids

**Figure 5:**
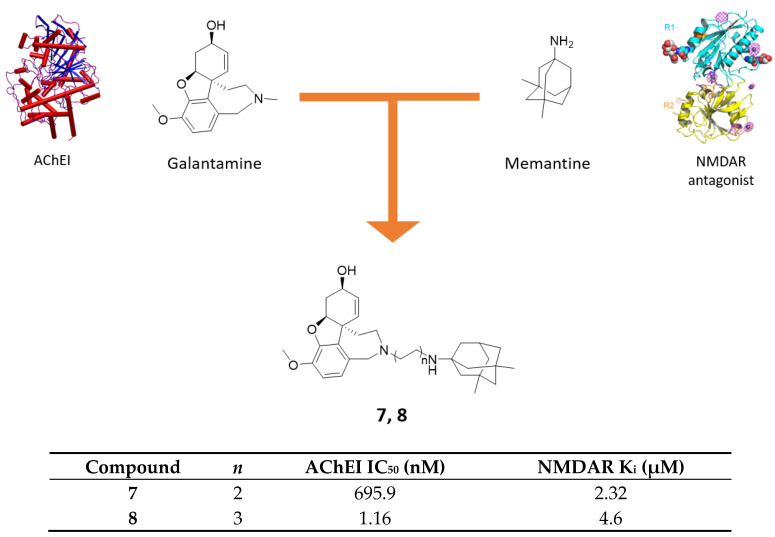
Drug design and biological activities of memantine-galantamine hybrids **7** and **8**.

Galantamine is an alkaloid-derived AChEI approved since 2001 for the treatment of mild-to-moderate forms of AD. Furthermore, galantamine associates AChEI efficacy to potentiation of NMDAR transmission mediated by protein kinase C (PKC) and to the ability of allosterically modulated α-subunit of nicotinic receptors [36,37]. Particularly, through the modulation of α7-nAChR on presynaptic glutamatergic neurons, it can enhance the beneficial effect of NMDAR’s synaptic activation, potentiating at the same time memantine’s neuroprotective effect [38]. These premises were further assessed in an in vitro validation assay where inactive dose of memantine and galantamine produced full neuroprotective efficacy, confirming promising experimental efficacy of their combination therapy [39]. In this context, after a computationally-driven SAR campaign, a series of galantamine-memantine conjugates, connected through the nitrogen atom of both, were developed, demonstrating to exert both NMDAR antagonism and AChE inhibition with high and well-balanced potencies [40]. It is important to note that for AChE inhibition, it is fundamental to identify the proper linker length between the two moieties, allowing interaction both to AChE’s CAS and PAS. In fact, 4-methylene linker of **7** prevented it from properly interacting with the PAS (identified with Trp286), thereby reducing its affinity when compared to derivatives with longer alkyl chains (e.g., **8**, Figure 5). Concurrently, for NMDAR antagonism, alkyl substituents on memantine’s nitrogen are allowed. In terms of AChEI activity, different lengths of the alkyl linker or N-methyl substitutions on memantine moiety are well tolerated, whereas spacer with heteroatoms and switching amine function of memantine into amide greatly affected AChEI profile. Regarding NMDAR, only alkyl or alkylamino linkers were allowed, whereas all other modifications were detrimental for NMDAR affinity.

Derivative **8** stood out as the best of the series regarding the AChEI/NMDAR profile as well as efficient NR2B binder (NMDAR with 2B subunit, peculiar of eNMDARs involved in glutamatergic overexcitation) and valid neuroprotective agent after insult by NMDA in SHSY-5Y cells (IC_50_ = 0.28 nM, Figure 5) [40]. Compound **7** was selected for further in vivo biological evaluation as a result of best compromise between well-balanced pharmacological potencies and physico-chemical properties (Figure 5) [41]. Due to a suboptimal pharmacokinetic profile, it was tested intracerebroventricularly in AD mouse model through osmotic minipump. After 7 days treatment at 7.5 µg/day, compound **7** was able to completely revert the induced neurotoxicity in vivo in terms of behavioral readouts (e.g., neuroprotective effect for short-term memory) and biochemical analysis (e.g., strong reduction of all neurodegenerative-related, apoptotic and synaptic plasticity biomarker) [41]. More recently, preliminary in vivo studies were conducted with compound **7** by transdermal iontophoresis delivery, looking to maintain the same pharmacological effects with a more patient-friendly administration. For now, compound **7** has proved to be delivered consistently to the brain through iontophoresis but further preclinical studies and pharmacokinetic-pharmacodynamic optimization are needed to prove its biological effects with this new innovative delivery system [42].

### 2.3. Aminoadamantane-Carbazole/Tetrahydrocarbazole Hybrids

It has been reported that some carbazole derivatives are potential AD-modifying compounds, i.e., *N*-alkyl carbazoles are able to favor the formation of soluble form of Aβ, through different mechanisms [43] and are also able to block apoptosis, thus exerting a neuroprotective effect [44]. Moreover, recent studies suggested that some carbazole-based compounds are valuable anti-AD drug candidates, since they are able to inhibit both AChE and BChE activity [45] as well as AChE- and self-induced Aβ aggregation [46]. These findings support the rationale to develop novel hybrids with a multimodal profile by adding to biological properties of the carbazole core the neuroprotection derived by the memantine nucleus.

**Figure 6:**
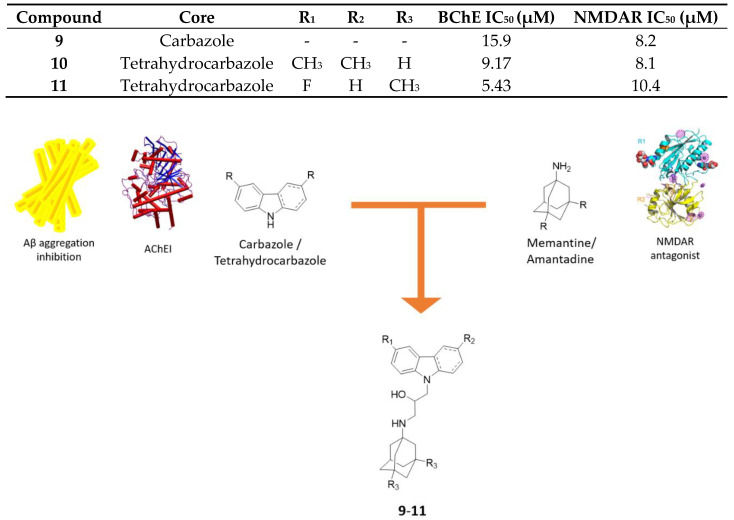
Drug design and biological activities of carbazole/tetrahydrocarbazole-aminoadamantane hybrids **9**–**11**.

Hybrids of aminoadamantanes with carbazole and tetrahydrocarbazole (Figure 6) were synthesized and tested as inhibitors of *h*AChE and equine serum BChE, as antagonists of NMDARs and as stimulators of microtubule assembly [47]. In AD, microtubule structure disturbance and tubulin polymerization suppression are signs of the incoming neurodegeneration that lead to failures in the axonal transport; therefore, it is necessary to develop compounds that are able to stabilize microtubule structure and to enhance tubulin assembly [47,48].

All the hybrids act as weak inhibitors of *h*AChE but are more potent and more selective for BChE and their inhibition activity is generally higher than reference carbazole (IC_50_ = 5–20 µM vs. >20 µM). The substitution of a carbazole moiety with a tetrahydrocarbazole fragment leads to an enhanced inhibition for BChE, while minimal differences between memantine and amantadine moiety have been observed. Overall, the highest inhibition activity for BChE is showed by compound **11** with a fluorinated tetrahydrocarbazole core. At the same time these compounds were tested as antagonists of NMDA receptors, and their ability to bind to the intrachannel (or MK-801 binding) site, present in all NMDARs, and/or to the allosteric (or ifenprodil-binding) site of NR2B-containing NMDARs, was assessed. The NR2B subunit is of particular interest because its blockade is beneficial for neuroprotection and Aβ-induced neuronal disruption [49].

In the carbazole series, compounds show different activities towards the allosteric site, and this feature depends primarily on the substitution patterns of the carbazole moiety: Indeed, compound **9** with no substituents is the most active. However, in the tetrahydrocarbazole series, this trend is not observed, in fact all the compounds are differently active towards both binding sites, except for compound **10**, which is selective towards the allosteric site of the NR2B subunit and also exhibits the most balanced multimodal profile. These heterodimers have substantially lower IC_50_ values than memantine and amantadine.

In both series, neither memantine moiety nor amantadine one seems important to stimulate tubulin polymerization, while the insertion of a chlorine or a bromine in the carbazole causes a reduction of this stimulating effect. Furthermore, the introduction of a fluorine in the tetrahydrocarbazole core and the unsubstitution of the carbazole one considerably enhances this effect, hence the most active compounds are the fluorinated tetrahydrocarbazole-memantine conjugate and the carbazole-memantine derivative. The fluorine-containing tetrahydrocarbazole hybrids reveal a high neuroprotection profile: Indeed, they are able to reduce the Ca^2+^-induced swelling of mitochondria and thus inhibit the mitochondria permeability transition (MPT), which is a key process in cell death, either in physiological conditions or in neurotoxicity models.

The same compounds were also evaluated and tested as hydrochlorides with compound **10** that showed the lowest IC_50_ value for the ifenprodil site [50]. Along with this, their effects were studied on the membrane potential of mitochondria, on the Ca^2+^ ion-caused depolarization and swelling of mitochondria, both indicating MPT. These novel compounds depolarize the membrane, indicating that they are toxic; however, exceptions can be made for compounds containing a fluorine as R substituent, which causes just a slight depolarization potential and shows a dose-dependent-inhibiting effect on Ca^2+^ ion-induced mitochondrial swelling, thus increasing mitochondrial stability and being assessed as potential neuroprotectors [50]. Following evaluation of the activity and selectivity of these compounds, molecular docking studies were performed, in order to shed light on the binding poses. It was reported that memantine and amantadine lay in the cavity located at the active site gorge of BChE, and their positions were very stable, while the ones of the linker and of the carbazole moiety changed. These compounds have a chiral centre, but the binding poses of the two enantiomers are very similar, with the hydroxyl groups that establish hydrogen bonds with different amino acid residues depending on the *R* or *S* isomer. Taking into account the position of the entire ligand, it was highlighted that it does not vary when amantadine is replaced by memantine, and a similar behavior is observed with the switch from carbazole to tetrahydrocarbazole; however, when a halogen substituent is inserted, the carbazole core undergoes a flip to reduce the steric strain [47].

These studies demonstrate that also carbazoles linked to memantine are able to maintain important NMDAR-blocking activities while adding interesting neuroprotection effects through tackling several pathological pathways involved in AD.

## 3. Memantine-Antioxidant Hybrids to Explore Neuroinflammation

In light of the above described multifaced nature of AD, the multitarget approach could help to elucidate insights in the complex pathogenetic network triggering pathologic conditions. Beside glutamate-driven excitotoxicity, oxidative stress, misfolded proteins and neuroinflammation are only some of the actors which, playing in liaison, define AD-impaired neuroenvironment. Particularly, prolonged eNMDAR activation leads to increasing cytoplasmic Ca^2+^ concentration and the onset of oxidative damage, which can in turn pull the trigger of aberrant misfolded protein cascade (e.g., amyloid plaques formation) [11]. Amyloid plaques are one of the classical trademarks of AD, characterized by Aβ_42_ deposition, which originates from the sequential cleavage of APP by β- and γ-secretases rather than the non-amyloidogenic and physiologic α-secretase cleavage. In concert, Aβ deposition triggered by excitotoxicity-leading oxidative stress can exacerbate oxidative damage in an etiopathogenetic loop, which involves microglia activation and the following neuroinflammatory conditions [14,51].

Based on these premises, linking memantine with bioactive payload able to tackle Aβ/ROS/neuroinflammation could be a promising pathway to deepen knowledge in NMDAR-mediated neurotoxic events in AD.

### 3.1. Memantine-Ferulic Acid Hybrids

**Figure 7:**
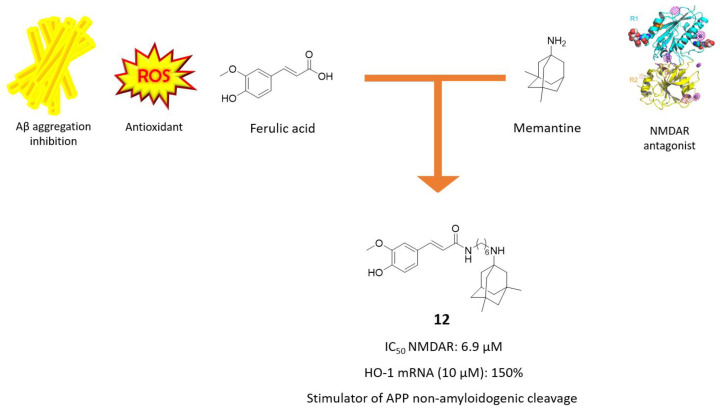
Drug design and biological activities of ferulic acid-memantine hybrid **12**.

Recently, Rosini and coworkers developed a series of hybrids between memantine and ferulic acid (FA), envisioned by the concurrent ability to reduce Aβ-induced neurotoxicity and oxidative stress of this latter [52,53]. The best derivatives of the series are those directly linked at the carboxylic function of FA and nitrogen atom of memantine through alkyl chain of different lengths, which were initially screened for their NMDAR antagonism efficacy. Derivative **12** came out as the most promising NMDAR-blocking agent, with potency only 3-fold less than memantine (IC_50_ = 6.9 µM vs. 2.3 µM, Figure 7), sharing with the parent compound the same open channel blocking mechanism and the binding site midway through the channel pore. Compound **12** exerted interesting antioxidant efficacy, both directly as radical scavenger and indirectly through activation of Nrf2-ARE pathway. Interestingly, differently from memantine and FA alone, it was also able to stimulate the non-amyloidogenic pathway and consequently limiting Aβ production at 10 µM concentration [53]. This new balanced memantine-hybrid, acting at a different level of AD pathological cascade, could be a starting point for further evaluation in more complex AD models, to deepen our findings in NMDAR-guided oxidative damage.

### 3.2. Memantine-Glutathione/Lipoic Acid Hybrids

**Figure 8:**
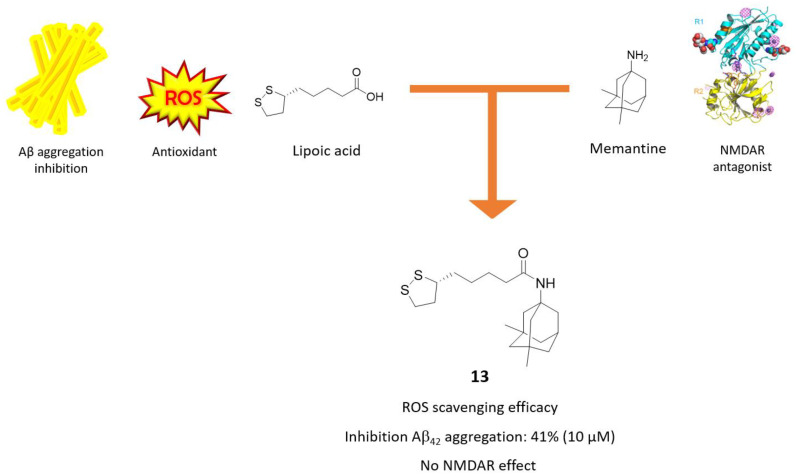
Drug design and biological activities of lipoic acid-memantine hybrid **13**.

The possibility of connecting antioxidant agents with the memantine core has also been explored [53,54] using radical scavengers such as glutathione (GSH) and (R)-α-Lipoic Acid (LA). They are endogenous molecules capable of removing free radicals that accumulate in cells, thus maintaining ROS balance at physiological levels and preventing oxidative stress and cell damage [55,56]. Furthermore, LA has been shown to mitigate Aβ aggregation and to protect from Aβ-mediated citotoxicity in vitro [57]. In light of these findings, Sozio, P. and coworkers developed conjugates of memantine linked to GSH and LA through an amide bond and tested them as NMDAR antagonists, Aβ aggregation inhibitors and as radical scavengers, pointing out compound **13** as the best derivative (Figure 8) [58].

The antioxidant properties of compound **13** were assessed in GL15 cell line upon the addition of hydrogen peroxide (H_2_O_2_) and superoxide radical anion (O_2_^−^); compared to LA, it showed a similar antioxidant activity towards H_2_O_2_, while a slightly lower one was shown towards O_2_^−^. In addition, compound **13** displayed Aβ antiaggregating properties (41% at 10 µM).

In order to investigate the effects of these conjugates on NMDAR, the authors used an in vitro protocol evaluating the release of [^3^H]Noradrenaline ([^3^H]NA) modulated by presynaptic NMDAR from rat hippocampal synaptosomes. Compound **13** turned out to be inactive as NMDAR ligand. This is consistent with previous SAR studies on memantine, which reported a loss of NMDAR activity upon conversion of the free adamantane’s amine group into amide group [40].

### 3.3. Amantadine-Propargylamine Hybrids

Monoamine oxidase B (MAO-B) with monoamine oxidase A (MAO-A) belong to flavoenzyme family bound to outer mitochondrial membrane. They exert a pivotal role in neurotransmission, regulating neurotransmitter metabolism and cellular redox balance. Particularly, MAO-B has been gaining particular interest in recent years due to its overexpression in neuroinflammatory condition and the consequential ROS overproduction and neurotransmitters impairment [59,60]. Accordingly, development of MAO-B inhibitors seems to be an interesting strategy to deepen our knowledge on diseases characterized by neuroinflammatory conditions, like AD and Parkinson disease (PD) [59].

**Figure 9:**
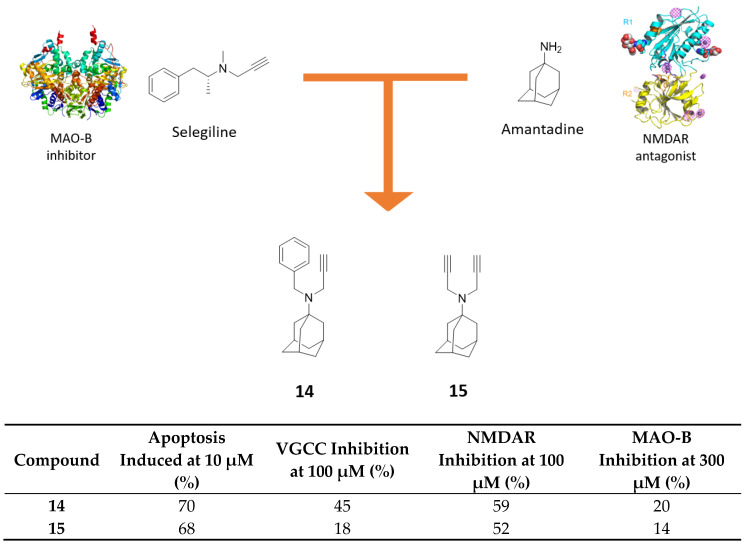
Drug design and biological activities of propargyl-amantadine hybrids **14** and **15**.

In 2014, Malan and coworkers developed a series of polycyclic propargylamine or acetylene derivatives with the aim to synergistically limit apoptotic processes and excitotoxicity, due to aberrant intracellular Ca^2+^ concentration and oxidative damage deriving from increasing MAO-B activity in AD. Polycyclic cores, such as memantine and amantadine, are established Ca^2+^ channel blockers, acting on NMDAR, while bigger polycyclic nucleus exert their effect, modulating *L*-type calcium channels [61]. In parallel, propargylamine moiety is the responsible fragment of MAO-B irreversible inhibition in compounds like selegiline, a drug used for the treatment of PD. Amantadine-propargylamine hybrids **14** and **15**, bearing respectively one or two propargyl functions directly connected to the nitrogen atom, demonstrated anti-apoptotic activity similar to selegiline (Figure 9) [62]. Compound **14** exhibited the best *L*-type voltage-gated calcium channels (VGCC) inhibitory activity (45% at 100 μM), whereas with compound **15** there was a drop of activity (18% at 100 μM), demonstrating the importance of N-benzyl substitution for target recognition. Both compounds maintained mild NMDAR inhibitory activity (52% for **15** and 59% for **14** at 100 μM) thanks to the presence of an amantadine core. Unfortunately, none of them exerted acceptable MAO-B inhibitory potency at 300 μM. These derivatives confirm the ability of memantine/amantadine scaffold to partially maintain NMDAR inhibitory activity, also in *N*-substituted derivatives (e.g., as previously seen in ferulic and galantamine hybrids), whereas it does not seem a good starting scaffold to reach satisfactory MAO-B inhibition.

## 4. Miscellaneous Memantine Derivatives

In this section, we review studies reported in the recent chemical and biological literature on the miscellaneous memantine derivatives for which AD neuroprotective activity derived by merging two pharmacophores is claimed, but which lie outside the hybrid series described in the other paragraphs of this review. In this regard, a lot of efforts have been made over the years to enhance memantine potency, synthesizing double-acting prodrug or targeting the second region of the same receptor to increase its efficacy [6,63]. Otherwise, merging two well-known NMDAR antagonists has been exploited as a strategy to obtain more efficient channel blockers with potential further clinical applications [64]. Furthermore, linking memantine to a purinergic receptor blocker has been pursued as a new approach to investigate other pathways involved in AD [65].

### 4.1. Memantine-Polyamine Conjugates

**Figure 10:**
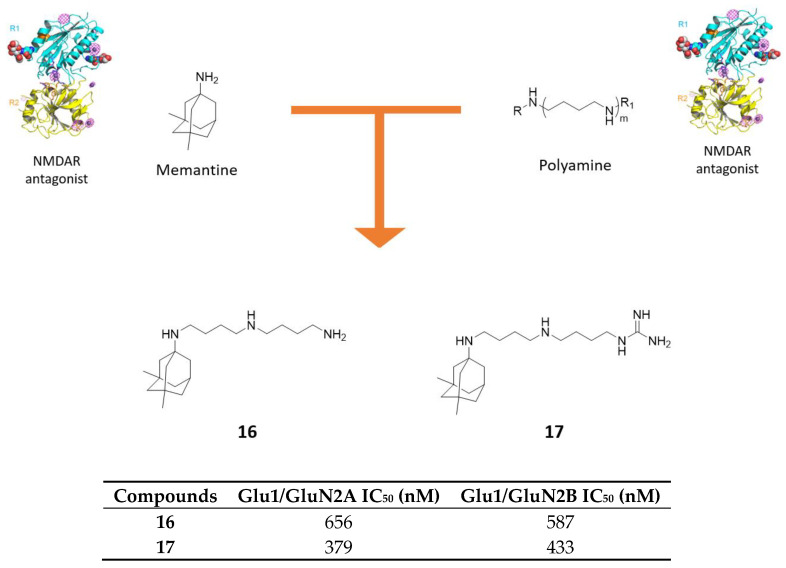
Drug design and biological activities of polyamine-memantine hybrids **16** and **17**.

Kumamoto, T. and coworkers developed a series of memantine-polyamine conjugates showing very potent NMDAR antagonism, higher than memantine [64]. The rationale is based on the well-known potent blocking NMDAR activity of the polyamine backbone, that the same authors have already conjugated with polycyclic scaffolds, developing compounds endowed with proved strong reversible and voltage-dependent NMDA blocking activity [66,67,68]. These derivatives were further explored and optimized, merging polyamine moiety with memantine scaffold (Figure 10). The triamine **16** and guanidino-diamine **17** demonstrated the highest inhibitory activities against GluN1/N2A and GluN1/N2B NMDAR (Figure 10). Particularly, compound **17** exhibited IC_50_ 3-fold lower against both receptors when compared with parent compound memantine (IC_50_ = 379 nM and 433 nM vs. 1376 nM and 2099 nM, respectively) [64].

### 4.2. H_2_S-Releasing Memantine Prodrug

**Figure 11:**
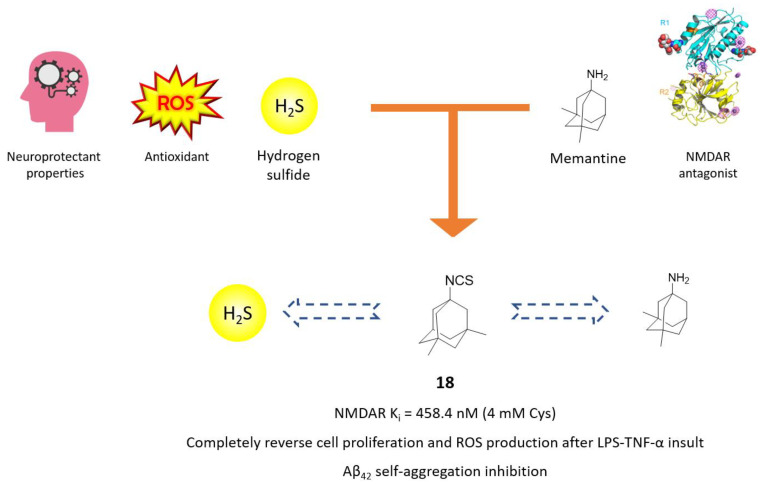
Drug design and biological activities of memantine prodrug **18**.

In recent years, the possibility of linking moieties releasing endogenous gaseous signalling molecules, i.e., gasotransmitters, with a well-known pharmacophore, has assumed particular interest in multitarget drug design [69,70]. Gasotransmitters, such as carbon monoxide (CO), nitric oxide (NO) and hydrogen sulfyde (H_2_S), are endogenously produced by enzymes and play important roles as antioxidants, neuromodulators and antinflammatory agents [71]. Particularly, H_2_S combines neuroprotection, antinflammatory and antiapoptotic activities to NMDAR-modulation ability both directly, with sulfhydration of critical cysteine residues, and indirectly, through regulation of intracellular calcium concentration [72,73]. On this basis, the interesting biological outputs of compound **18**, an H_2_S-releasing memantine derivative by Sestito and coworkers, can open a range of possibilities for new MTDL campaign [63]. Compound **18** bears an isothiocyanate moiety as H_2_S-donating moiety, replacing the characteristic free amino group of memantine (Figure 11). First of all, compound **18** showed to maintain memantine’s NMDAR inhibition (K_i_ = 458 vs. 329 nM) only in the presence of 4 mM *L*-cysteine, which can mediate H_2_S release and memantine’s unveiling. Amperometric analysis on derivative **18** demonstrated a prolonged and persisting cysteine-mediated H_2_S releasing. Furthermore, compound **18** was able to protect cells from oxidative stress and LPS-TNFα insult, reducing ROS production and stimulating cell proliferation in neuroinflammation in an in vitro model at 10 μM (Figure 11). Antiamyloidogenic properties of memantine were confirmed with compound **18**, which demonstrated to reduce Aβ_42_ self-induced aggregation and to reduce Aβ-induced damage in rat microglia cells to roughly the same extent as its parent compound [63].

### 4.3. Dual P2X7-NMDA Receptor Antagonists

P2X7 receptor (P2X7R) is an ATP-sensitive ion channel, present in the CNS (astrocytes, oligodendrocytes and microglia), where it mediates K^+^ efflux and Ca^2+^/Na^+^ influx [74,75]. In physiological conditions, it exerts a neuroprotective effect, i.e., the activation of the cAMP response element-binding (CREB), which inhibits the transcription of genes involved in the microglial inflammatory response [76] and the stimulation of α-secretase, which is responsible for the non-amyloidogenic cleavage of the amyloid precursor protein (APP), with the formation of soluble and non-toxic sAPPα [77].

In the presence of high ATP levels, like in AD, microglial P2X7Rs are upregulated, giving rise to an inflammatory cascade, where IL-1β, INF-γ, TNF-α, and other cytokines are released, and this contributes to the progression of the disease [74,78,79]. Furthermore, P2X7Rs release glutamate [80], which activates NMDARs [81]. A research conducted on AD mouse model reported that the in vivo P2X7R inhibition reduced the levels of Aβ, thus showing a neuroprotective effect. However, the effects of P2X7R inhibition in pathological conditions are still poorly understood and need further investigation [78].

**Figure 12:**
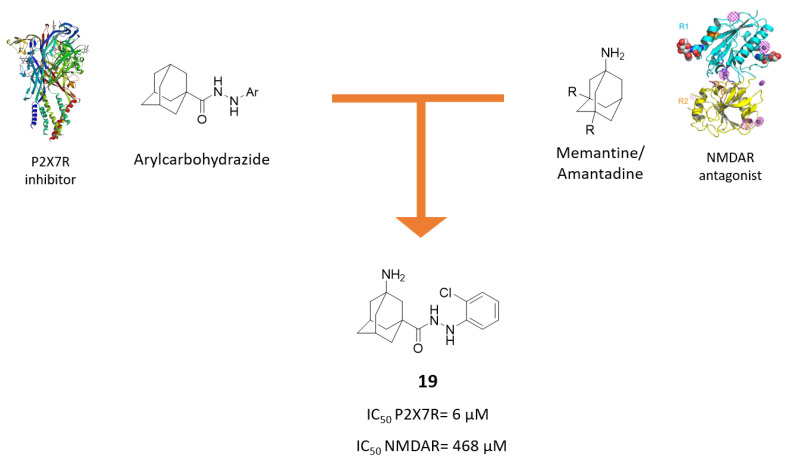
Drug design and biological activities of N’-arylcarbohydrazide-aminoadamantane hybrid **19**.

Based on these premises, new P2X7R-NMDAR antagonists were developed based on an amantadine or memantine moiety bound to a N’-arylcarbohydrazide core, which is correlated to strong P2X7R antagonism (Figure 12) [65].

Compound **19** bearing a *o*-chloro phenyl ring showed the highest inhibitory activity towards P2X7R, but lower blocker activity towards NMDAR compared to amantadine. Hence, following the encouraging P2X7 inhibition, structural modifications were made to improve NMDAR antagonism by replacing the amantadine core of compound **19** with a memantine moiety. However, the resulting hybrids were not able to significantly inhibit NMDARs and also showed higher IC_50_ values for P2X7R than compound **19**.

The study demonstrates that these molecules could potentially slow down neuroinflammation by preventing the P2X7R-induced inflammatory cascade. Even if they effectively bind to the P2X7R, they did not show an improved activity for NMDAR compared to memantine.

## 5. Conclusions and Future Perspectives

Although AD current pharmacotherapy is founded on cholinergic and glutamatergic hypotheses, the drugs licensed for AD treatment acting as AChEIs or as NMDAR antagonist (memantine) provide only symptomatic and temporary support. In recent years, the research of new therapeutic agents has been mainly centred on the amyloidogenic hypothesis and based on the multifactorial nature of AD. The assumption that intractable neurodegenerative diseases like AD are caused by more than one interacting mechanism has driven many research groups in designing and synthesising small molecules by merging two or more distinct pharmacophores with different biological properties. In this context, memantine represents a privileged structure in the design of a variety of multifunctional compounds to be developed as new AD therapeutic agents. In this review, we have reported the more recent studies relative to the molecular hybridization of memantine or its amantadine nucleus with the chemical features of AChEI (tacrine and galantamine), on the basis of the close interplay between the glutamatergic and the cholinergic systems in disease process, and with MAO inhibitor (propargylamine) and neuroprotectant (ferulic and lipoic acid, and carbazole) scaffolds with the aim to halt the etiopathogenetic loop of neuroinflammation involving glutamatergic excitotoxicity, oxidative stress and protein aggregation. The biological profile of the new molecules, in some cases very promising, highlights the great challenge for medicinal chemists in finding disease-modifying agents with balanced activities towards the different targets involved in AD. Several issues remain to be addressed to translate AD multitarget approaches into effective drug candidates against AD, mainly a better comprehension of disease processes and a higher predictive validity of animal models.
